# Phase 2 study of chidamide in combination with CAG and venetoclax-azacitidine in older patients with newly diagnosed acute myeloid leukemia

**DOI:** 10.3389/fimmu.2025.1525110

**Published:** 2025-02-26

**Authors:** Qingyang Liu, Jingjing Yang, Lei Lv, Xiawei Zhang, Meng Li, Lingmin Xu, Sai Huang, Yu Jing, Liping Dou

**Affiliations:** ^1^ State Key Laboratory of Experimental Hematology, Senior Department of Hematology, The Fifth Medical Center of Chinese PLA General Hospital, Beijing, China; ^2^ Medical School of Chinese PLA, Beijing, China

**Keywords:** venetoclax, azacitidine, CACAG-VEN regimen, older patients, acute myeloid leukemia

## Abstract

**Introduction:**

Older patients with acute myeloid leukemia (AML) respond poorly to standard induction therapy. DNA methyltransferases (DNMTs) and histone-deacetylases (HDACs) are key regulators of gene expression in cells and have been investigated as important therapeutic targets. However, their effects remains unclear as induction therapy for AML.

**Methods:**

Previously untreated AML patients aged 60 years and over (N=40) were enrolled into this single arm, open-label, phase 2 study to evaluate the clinical efficacy and safety of chidamide combined with CAG and venetoclax-azacitidine (referred to as CACAG-VEN) in elderly AML patients (ClinicalTrials.gov:NCT05659992). All patients received induction treatment with aclarubicin (10 mg/m2/d on days 1, 3, and 5), azacitidine (75 mg/m2 on days 1–7), cytarabine (75 mg/m2 bid on days 1–5), chidamide (30 mg, twice/week for 2 weeks), and venetoclax (100 mg on day 1, 200 mg on day 2, 400 mg on days 3–14). Granulocyte colony-stimulating factor 5 mg/kg/day was administered.

**Results:**

Theoverall response rate was 97.5%, with a composite complete response (CRc) rate of 85.0% after one cycle of CACAG-VEN. Patients with adverse risk according to the ELN guidelines had CRc rates of 81.3%. No patients experienced early death within 30 days of therapy initiation. Grade 3 - 4 non-hematological adverse events included febrile neutropenia in 15 (37.5%) of 40 patients, pneumonia in three (7.5%), sepsis in two (5.0%) and blood bilirubin increase in one (2.5%). The 12-month overall survival rate was 73.4% (95% CI: 55.9–84.8%). The median time to recovery was 15.0 (IQR 10.0-19.5) days for platelets ≥ 20000/mL and 13.0 (IQR 10.5-17.0) days for an absolute neutrophil count ≥ 1000 cells/mL after induction therapy.

**Discussion:**

In conclusion, chidamide in combination with CAG and venetoclaxazacitidine was effective and well tolerated in elderly patients with AML.

**Clinical trial registration:**

https://www.clinicaltrials.gov/, identifier NCT05659992.

## Introduction

Older adults account for most cases, with a median age of 68 years at diagnosis (1). Elderly patients with AML often respond poorly to induction chemotherapy as a result of biological disease-related factors such as increased frequency of adverse-risk cytogenetic and molecular features, secondary acute myeloid leukemia, and increased expression of multidrug resistance phenotypes (2, 3). Furthermore, because of poor performance status, comorbidities, and reduced organ function, older patients may not be candidates for conventional cytotoxic induction therapies (4–6). Thus, a crucial need exists to develop more effective, well tolerated therapies for elderly patients with acute myeloid leukemia.

With the development of epigenetic studies, a growing body of research has shown that epigenetic modifications play a crucial role in the development of chemoresistance. DNA methyltransferases (DNMTs) and histone-deacetylases (HDACs) are major epigenetic mediators and can be pharmacologically reversed by DNMT inhibitors or HDAC inhibitors. These agents include HDAC inhibitors, such as chidamide, vorinostat, and romidepsin, and DNM inhibitors, including azacitidine and decitabine. A previous study investigated DNMTi, cytarabine, aclarubicin, and G-CSF (DCAG) in the induction treatment for patients aged from 55 to 69 years old with newly diagnosed AML. In this study, patients in the DCAG group achieved similar overall response (ORR), complete remission (CR), overall survival (OS) and relapse-free survival (RFS) as those who received the “3 + 7” regimen. Notably, patients exhibited better tolerance to the DCAG regimen (7). HDACs are key regulators of gene expression in cells and have been investigated as important therapeutic targets for cancer and other diseases (8, 9). Chidamide is the first oral selective HDAC inhibitor for HDAC1, HDAC2, HDAC3, and HDAC10 and is likely to potentiate the sensitivity of cancer cells through the expansion of existing drug-binding sites and the establishment of novel interaction sites (10, 11). In this study, chidamide was incorporated into the DCAG regimen with the aim of enhancing the response rate in patients with AML.

The anti-apoptotic protein B-cell lymphoma 2 (BCL-2) is highly expressed in leukemia stem cells and is linked to chemotherapy resistance and poor prognosis in acute myeloid leukemia patients (12, 13). BCL-2 maintains myeloblast survival by binding and inhibiting the pro-apoptotic protein BAX, leading to mitochondrial reliance on BCL-2. BAX is released when BCL-2 is antagonized, causing mitochondrial outer membrane permeabilization and triggering cell death (14, 15). Venetoclax, a potent and selective oral BCL-2 inhibitor, has shown clinical efficacy as a monotherapy with a manageable safety profile in patients with relapsed or refractory AML (16). Venetoclax cannot precisely attack tumor cells but enhances anti-tumor effect of anthracyclines through the apoptosis. Therefore, it is crucial to combine venetoclax with other drugs (17). Venetoclax in combination with azacitidine has demonstrated a synergistic effect in preclinical models of AML cells (18). Venetoclax combined with low-dose cytarabine or demethylating drugs has been approved for older patients with newly diagnosed AML (19). With this rationale, we conducted a single-arm phase 2 trial to investigate the safety and efficacy of venetoclax combined with chidamide, azacitidine, cytarabine, aclarubicin, and G-CSF as an induction treatment (referred to as CACAG-VEN) for older adults (aged ≥ 60 years) with newly diagnosed AML.

## Methods

### Patients and procedures

We conducted a single-center, non-randomized, open-label, phase 2 study (ClinicalTrials.gov: NCT05659992) at the Chinese PLA General Hospital. The study was approved by the Ethics Committee of the Chinese PLA General Hospital and was executed in strict adherence to the principles outlined in the Declaration of Helsinki. Eligible patients were aged ≥ 60 years with confirmed diagnosis of AML, excluding cases of acute promyelocytic leukemia (20, 21). All patients were treated at the Chinese PLA General Hospital between December 25, 2022 and June 5, 2024. A total of 40 patients were included in the study, and the calculation of the sample size was provided in **Supplementary Methods**. Detailed information about the criteria for patient inclusion and exclusion is presented in **Supplementary Table S1**.

All patients in this study were treated with the CACAG-VEN regimen over a 28-day cycle: intravenous aclarubicin (10 mg/m^2^, per day, on days 1, 3, and 5), subcutaneous azacitidine (75 mg/m^2^ on days 1-7), intravenous cytarabine (75 mg/m^2^ twice per day, on days 1-5), oral chidamide (30 mg, twice per week for 2 weeks), and oral venetoclax (100 mg on day 1, 200 mg on day 2, 400 mg on days 3–14). Granulocyte colony-stimulating factor (G-CSF) 5 μg/kg per day was administered until granulocyte recovery (**Supplementary Figure S1**, **Supplementary Methods**).

The sample size was calculated according to the primary endpoint (overall response, ORR) of the study. Anthracyclines combined with cytarabine is the classic regimen for acute myeloid leukemia (AML) treatment. Investigators at the M.D. Anderson Cancer Center analyzed data on 998 older patients aged 65 and older receiving intensive chemotherapy at their institution, the ORR after induction therapy with “3 + 7” regimen was reported to be 20%-50% (6). The ORR of venetoclax in combination with decitabine or azacitidine in treatment-native, elderly patients with AML were 68%. Therefore, in the sample calculation, we chosen 35% as the reference ratio value and determined that the expected ORR for patients received with venetoclax combined with chidamide, azacitidine, cytarabine, aclarubicin, and G-CSF as an induction treatment (referred to as CACAG-VEN) was 68% (22). This study was planned at a 2-sided significance level α= 5% with a power of 1-β = 80%. Twenty-eight patients were required for each group as estimated using Z-Test for single sample rate. Allowing a drop-out rate of 10%, a total of more than 30 patients were required (23, 24).

### Endpoints

The primary endpoint was the overall response rate after one cycle of induction (ORR, CR plus complete response with incomplete blood cell count recovery [CRi], plus partial response [PR]) according to the modified International Working Group response criteria for AML (25). The secondary endpoints were composite complete response (CRc, CR+CRi), MRD after one or two cycles of induction, 1-year duration of response (DOR), cumulative incidence of relapse (CIR), event-free survival (EFS), and OS. Treatment-related adverse events were defined as adverse events that occurred from the first dose of the study treatment to 30 days after the discontinuation of treatment (**Supplementary Methods**). The severity of adverse events was graded according to the Common Terminology Criteria for Adverse Events (CTCAE), version 5.0 (26).

### Statistics

Continuous data are described as the median with interquartile range (IQR) or mean and standard deviation according to the normality of the distribution. Categorical data are described as n (%). The Kaplan–Meier method was used to estimate the DOR, EFS, and OS. The cumulative incidence of relapse was estimated using a competing risk model. Death without relapse was defined as a competing event for relapse. Safety analysis was used to calculate the frequency of various events. Any difference for which the two-sided P < 0.05 was considered statistically significant. Statistical analyses were performed using environment R (version 4.1.2), SPSS (version 27.0), and GraphPad Prism software (version 10.1.2).

## Results

### Patient’s characteristics

**Figure 1:**
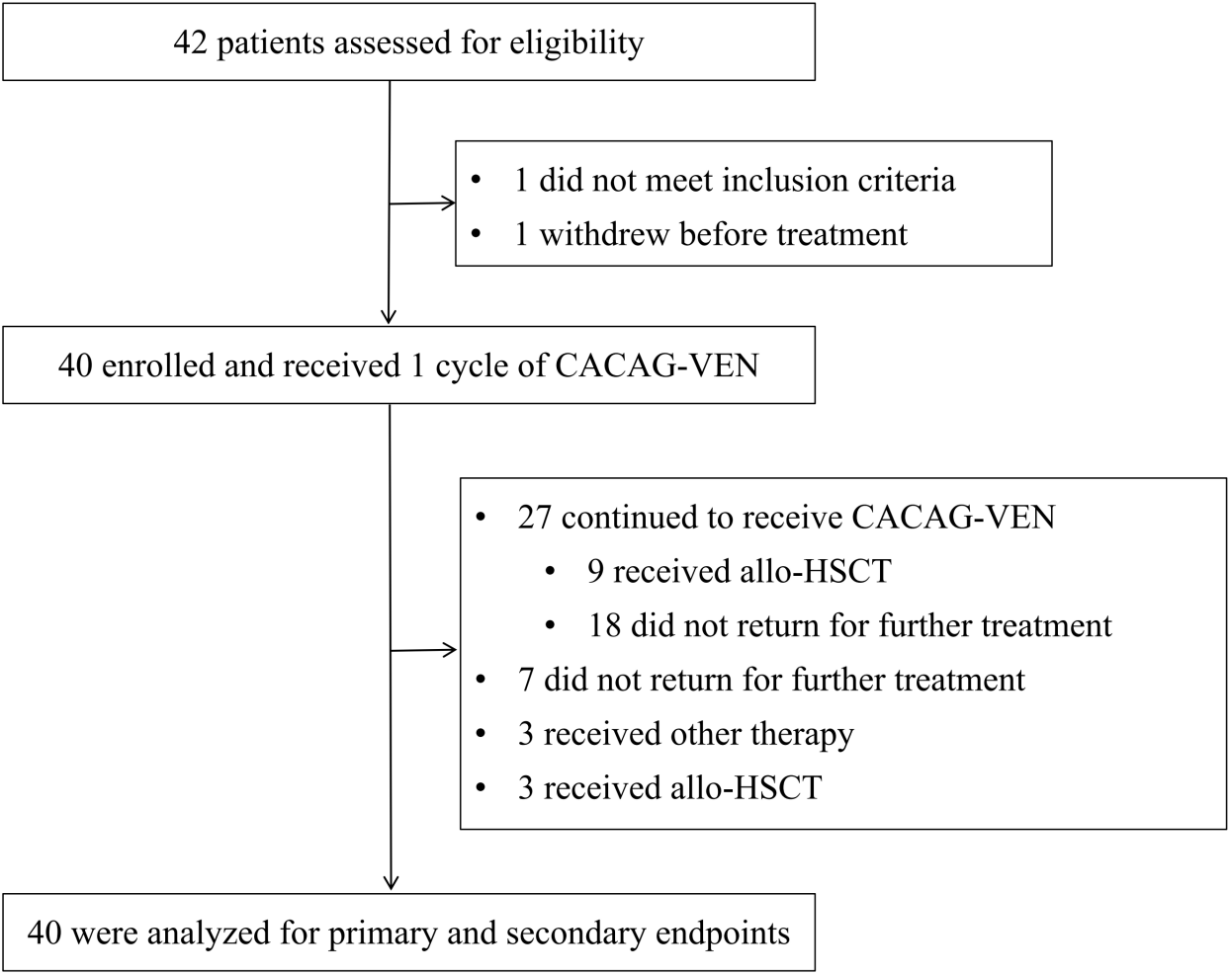
Trial profile. CACAG-VEN, venetoclax combined with chidamide, azacitidine, cytarabine, aclarubicin, and granulocyte colony-stimulating factor; other therapy, including VAC (venetoclax combined with chidamide and azacitidin), VAPC (venetoclax combined with chidamide, azacitidine, cytarabine, and camrelizumab), CACHG (azacitidine combined with chidamide, homoharringtonine, cytarabine, and granulocyte colony-stimulating factor); allo-HSCT, allogeneic hematopoietic stem cell transplantation.

**Figure 2:**
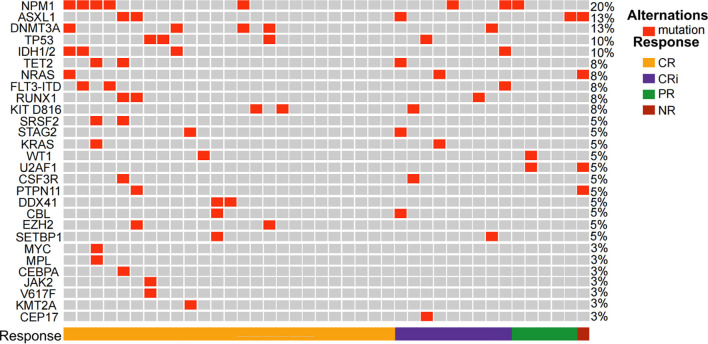
Heatmap of the study patients depicting mutations and overall response after the first cycle of CACAG+VEN regimen. CR, complete response; CRi, CR along with incomplete blood count recovery; PR, partial response; NR, no response; CACAG+VEN, venetoclax combined with chidamide, azacitidine, cytarabine, aclarubicin, and granulocyte colony-stimulating factor.

Between December 25, 2022, and June 5, 2024, 42 patients were screened for the study, of whom 40 were enrolled (**Figure 1**, **Supplementary Table S2**). Among the two excluded patients, one withdrew before treatment, and the other did not meet inclusion criteria. These patients were treated with the CACAG-VEN regimen. Baseline patient characteristics are summarized in **Table 1**. The median age was 64.0 years (range: 60.0–74.0 years), with 25 (62.5%) male patients. Thirty patients (75.0%) had *de novo* AML. Sixteen patients (40.0%) were categorized as adverse risk according to the ELN guidelines. Among the included patients, NPM1 was mutated in 20.0%, ASXL1 in 12.5%, DNMT3A in 12.5%, and TP53 in 10% of patients (**Figure 2**). All patients received at least one treatment with the CACAG-VEN regimen, 13 patients (32.5%) received only one cycle and 27 (67.5%) received the second cycle of the CACAG regimen. Twelve patients (30.0%) received allo-HSCT after chemotherapy.

**Table 1:** Patient demographics and clinical characteristics.

Characteristic	Patients (n=40)
Age, median (range) years	64.0 (60.0-74.0)
Gender, n (%)	
Male	25 (62.5)
AML type (n, %)	
*De novo*	30 (75.0%)
Secondary	10 (25.0%)
ECOG performance score, n (%)	
0	12 (30.0)
1	22 (55.0)
2	6 (15.0)
Baseline parameters	
WBC, 10^9^/L, median (range)	8.39 (1.22-176.4)
Hb, g/L, median (range)	80.5 (2.84-163.0)
Plt, 10^9^/L, median (range)	58.5 (18.0-289.0)
Bone marrow blast cell proportion at baseline	
Median (range)	52.5 (17.3-93.2)
< 30%, n (%)	10 (25.0)
≥ 30% ~ < 50%, n (%)	7 (17.5)
≥ 50%, n (%)	23 (57.5)
Karyotype	
Normal	39 (97.5)
Complex^*^	1 (2.5)
Cytogenetic/molecular Risk (ELN 2022)	
Favorable Risk	6 (15.0%)
Intermediate Risk	18 (45.0%)
Adverse Risk	16 (40.0%)
Selected molecular mutation	
NPM1	8 (20.0%)
ASXL1	5 (12.5%)
DNMT3A	5 (12.5%)
TP53	4 (10.0%)
IDH1/2	4 (10.0%)
TET2	3 (7.5%)
FLT3-ITD	3 (7.5%)
RUNX1	3 (7.5%)
NRAS	3 (7.5%)

ECOG, eastern cooperative oncology group; WBC, white blood cells; Hb, hemoglobin; Plt, platelets; ELN, European LeukemiaNet; Complex^*^, defined as a karyotype with three or more chromosomal abnormalities, without restriction on the type of abnormality.

### Clinical response

**Figure 3:**
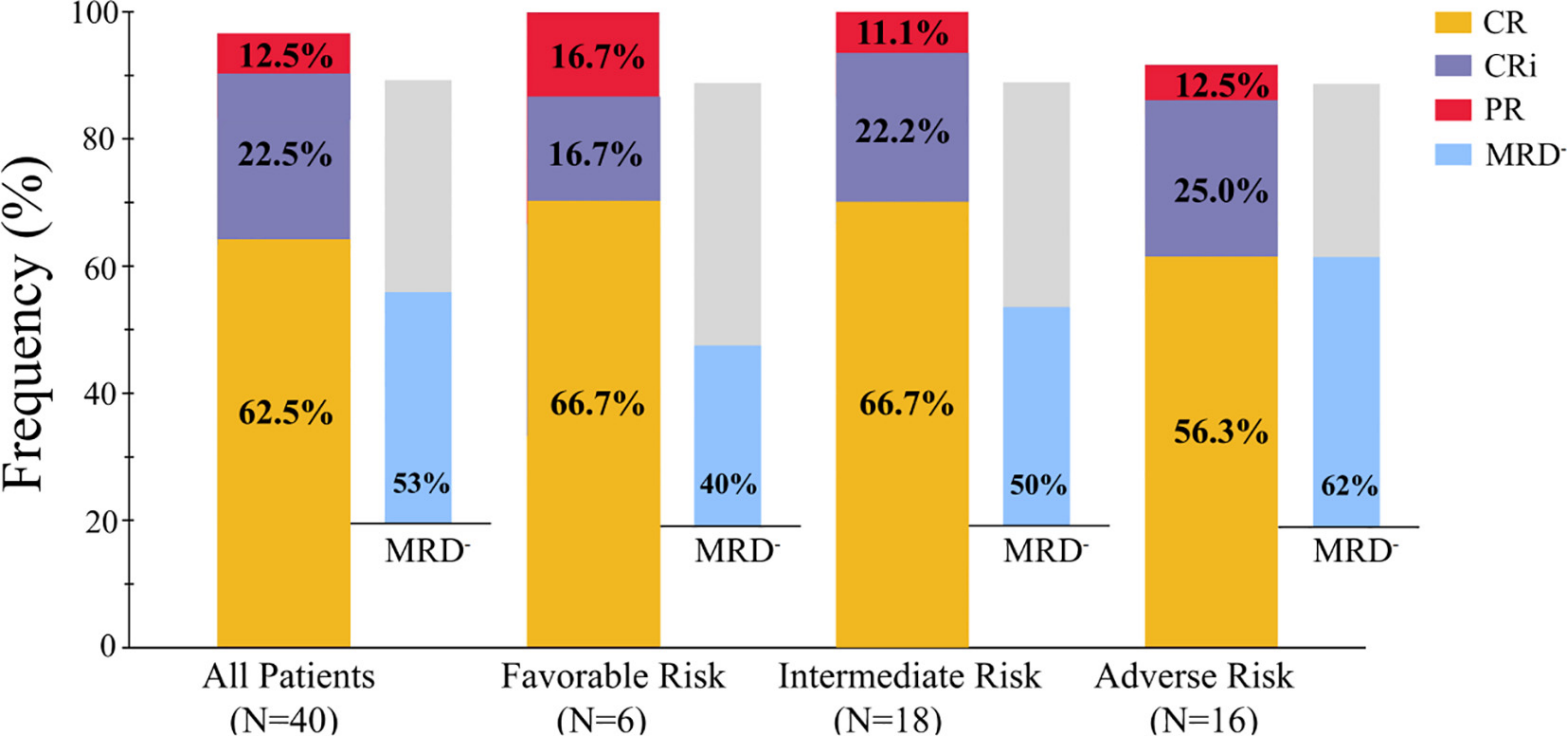
Overall response in patients received one cycle of therapy. CR, complete response; CRi, CR along with incomplete blood count recovery; PR, partial response; NR, no response; MRD, measurable residual disease.

All 40 enrolled patients completed the first course and were included in the response assessment. The ORR was 97.5% (39/40; 95% CI: 85.3–99.9) after one cycle of the regimen. The CRc rate was 85.0% (34/40; 95% CI: 69.5–93.8). Twenty-five patients (62.5%; 95% CI: 45.8–76.8) achieved CR, 9 patients (22.5%; 95% CI: 11.4–38.9) achieved CRi, and 5 patient (12.5%; 95% CI: 4.7–27.6) achieved PR (**Table 2**). One of the 40 patients did not respond to one cycle of CACAG-VEN therapy. This patient received the second cycle of CACAG-VEN for remission induction and achieved CR with an MRD-positive status, achieving MRD-negative status after allo-HSCT. The CRc rate was 83.3% (5/6; 95% CI: 36.5–99.1) in patients with ELN-favorable risk, 88.9% (16/18; 95% CI: 63.9–98.0) in patients with intermediate risk, and 81.3% (13/16; 95% CI: 54.0–95.0) in patients with adverse risk (**Table 2**, **Figure 3**). Among those who reached complete remission, measurable residual disease-negativity was reached in 52.9% (18/34, 95% CI: 35.4–69.8) of the total patients, 40.0% (2/5, 95% CI: 7.3–83.0) in the favorable risk group, 50.0% (8/16, 95% CI: 25.5–74.9) in the intermediate risk group, and 61.5% (8/13, 95% CI: 32.3–84.9) in the adverse risk group (**Table 2**). In the analysis of the molecular subgroups, patients with RUNX1, FLT3-ITD, or TET2 mutations exhibited CRc rates of 100% (3/3,95% CI: 31.0–100.0, **Figure 2**). The CRc rates for patients with DNMT3A, TP53, IDH1/2, NPM1, NRAS, and ASXL1 were 100.0% (5/5, 95% CI: 46.3–100.0), 100.0% (4/4, 95% CI: 39.6–100.0), 100.0% (4/4, 95% CI: 39.6–100.0), 87.5% (7/8, 95% CI: 46.7–99.3), 66.7% (3/4,95% CI: 12.5–98.2), and 60.0% (3/5, 95% CI: 17.0-92.7).

**Table 2:** Response after first cycle of the CACAG-VEN regimen (n= 40).

Overall response rate	Patients (n=40)	Patients (n=30)		
		Favourable Risk* (n=6)	Intermediate Risk* (n=18)	Adverse Risk* (n=16)
ORR % ( 95%CI)	97.5 (85.3-99.9)	100.0 (51.7-100.0)	100.0 (78.1-100.0)	93.8 (67.7-99.7)
CR or CRi, n (%, 95%CI)	34 (85.0, 69.5-93.8)	5 (83.3, 36.5-99.1)	16 (88.9, 63.9-98.0)	13 (81.3, 54.0-95.0)
CR, n (%, 95%CI)	25 (62.5, 45.8-76.8)	4 (66.7, 24.1-94.0)	12 (66.7, 41.1-85.7)	9 (56.3, 30.6-79.3)
CRi, n (%, 95%CI)	9(22.5, 11.4-38.9)	1 (16.7, 0.9-63.5)	4 (22.2, 7.3-48.1)	4 (25.0, 8.3-52.3)
PR, n (%, 95%CI)	5 (12.5, 4.7-27.6)	1 (16.7, 0.9-63.5)	2 (11.1, 2.0-36.0)	2 (12.5, 2.2-39.6)
NR, n (%, 95%CI)	1 (2.5, 0.0-14.7)	0 (0.0, 0.0-48.3)	0 (0.0, 0.0-21.8)	1 (6.3, 0.3-32.3)
MRD-negative rate in patients with response (95%CI)	52.9 (18/34, 35.4-69.8)	40.0 (2/5, 7.3-83.0)	50.0 (8/16, 25.5-74.5)	61.5 (8/13, 32.3-84.9)

CR, complete response; CRi, CR along with incomplete blood count recovery; PR, partial response; NR, no response; ORR, overall response; MRD, measurable residual disease. *, patients were stratified based on ELN (2022) risk assessment criteria; CACAG-VEN, venetoclax combined with chidamide, azacitidine, cytarabine, aclarubicin, and granulocyte colony-stimulating factor.

Following the first cycle, 7 patients refused further treatment. The remaining patients (n = 33) received subsequent therapy as follows: 27 received the second cycle of CACAG-VEN therapy, 3 underwent allo-HSCT after achieving CR, and 3 received other treatment. Among the patients (n=27) receiving the second cycle of the CACAG regimen, one patient dying before the bone marrow evaluation, the rate of CRc was 96.2% (CR: 53.9%, 14/26; CRi: 42.3%, 11/26, **Supplementary Table S3**). Notably, those receiving two cycle of CACAG-VEN regimen tended to exhibit a higher MRD-negativity rate than those receiving one cycle of CACAG-VEN regimen. (76.0% vs. 52.9%, **Supplementary Table S3**).

### Survival

**Figure 4:**
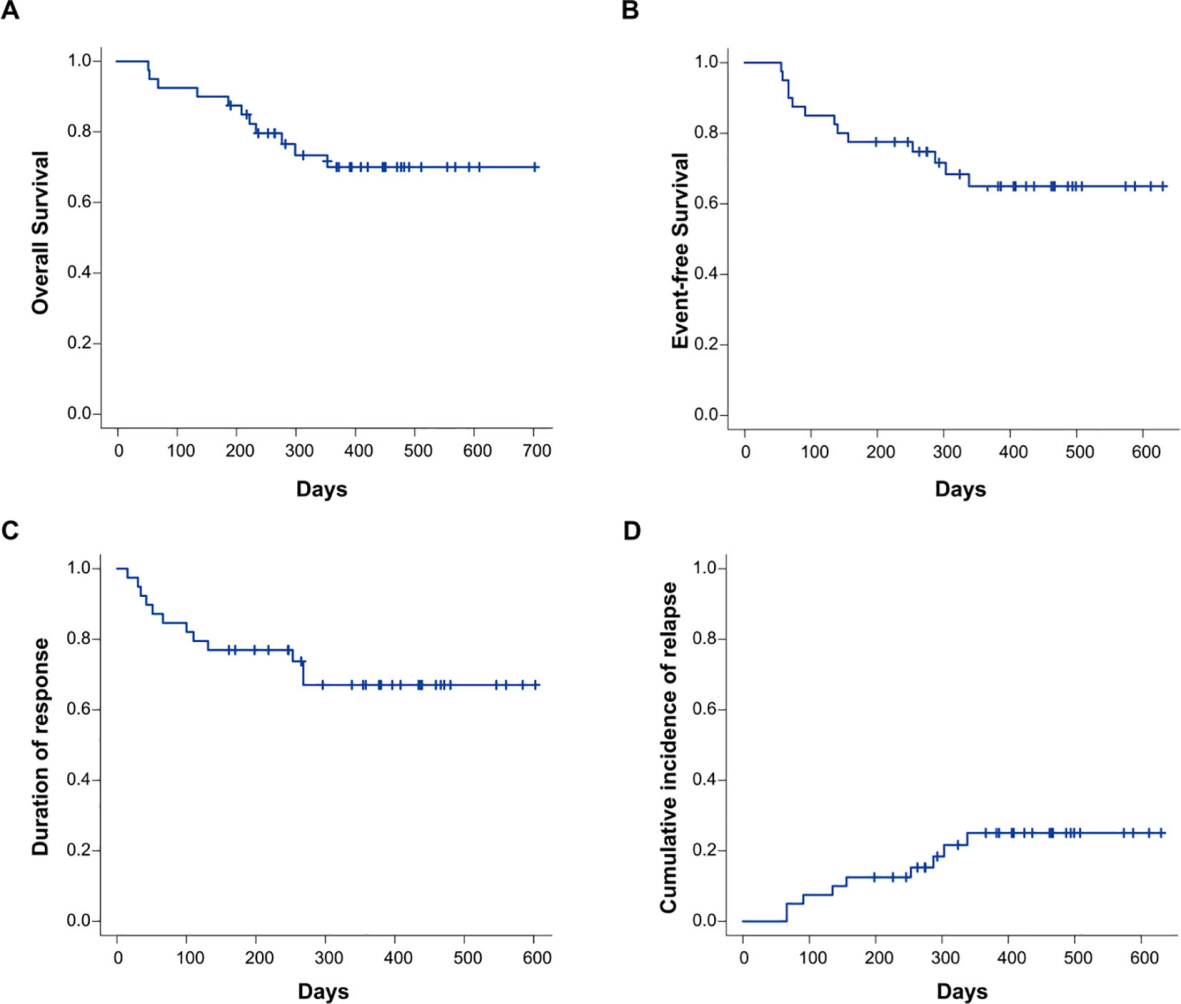
Cumulative incidence of OS **(A)**, EFS **(B)**, DOR **(C)**, and CIR **(D)** in the entire study cohort (n=40). OS, overall survival; EFS, event-free survival; DOR, duration of response; CIR, cumulative incidence of relapse.

The follow-up cutoff date was December 20, 2024.The median duration of follow-up was 462 days (198-726). No deaths occurred in AML patients within 30 days of protocol therapy. The OS rate at 12 months was 73.4% (95% CI: 55.9–84.8, **Figure 4A**). The 12-month EFS,DOR and CIR was 64.9% (95% CI: 47.0–78.1, **Figure 4B**), 67.0% (95% CI: 48.9–79.9, **Figure 4C**) and 25.1% (95% CI: 12.1–40.4, **Figure 4D**).

Among patients achieving CRc after one cycle of CACAG-VEN, survival outcomes were comparable in OS, EFS, and DOR between MRD-negative and -positive patients (OS at 12-month: 71.8% [MRD-] vs. 64.8% [MRD+], *P* = 0.81; EFS at 12-month: 66.2% [MRD-] vs. 65.6% [MRD+], *P* = 0.87; DOR at 12-month: 66.2% [MRD-] vs. 65.6% [MRD+], *P* = 0.91, **Supplementary Figures S3A–C**). For patients who achieved CRc after one cycle of CACAG-VEN, MRD-negative patients showed a trend of decreased CIR compared to MRD-positive patients, albeit with non-significant statistical differences between the groups. (CIR at 12-month: 17.1% [MRD-] vs.28.1% [MRD+], *P* = 0.51, **Supplementary Figure S3D**). Compared with patients who did not receive allo-HSCT, those who received allo-HSCT were comparable in OS and EFS. (OS at 12 months, 82.5% [allo-HSCT] vs. 69.4% [non-HSCT], *P* = 0.75; EFS at 12 months, 53.5% [allo-HSCT] vs. 65.9% [non-HSCT], *P* = 0.98, **Supplementary Figures S4A–B**).

### Safety

Overall, this regimen was overall tolerable, with all patients completing treatment without dose reduction. No patients experienced early death within 30 days of therapy initiation. No grade 5 adverse events were observed. The most common grade 3–4 non-hematological adverse events were febrile neutropenia (15 [37.5%] of 40 patients), pneumonia (three [7.5%]), sepsis (two [5.0%]) and blood bilirubin increase (one [2.5%], **Table 3**). No venous thrombosis or tumor lysis syndrome events were observed. The most common grade 3–4 hematological toxicities were neutropenia (40 [100.0%] patients), thrombocytopenia (40 [100.0%]), and anemia (40 [100.0%], **Table 3**). No treatment-related deaths occurred. The median time to recovery of an absolute neutrophil count of 1000 or more cells per μL was 15.0 (IQR 10.0-19.5) days. The median time to recovery of a platelet count of 20000 or more platelets per μL was 13.0 (IQR 10.5-17.0) days (**Supplementary Table S4**). Packed red cells and platelets transfused for patients were 8.5 (range: 2.0–23.5) units and 8.5 (range: 2.0–19.5) units.

**Table 3:** Adverse event during first cycle of therapy (n=40).

	Grade 1	Grade 2	Grade 3	Grade 4	Total, n(%)
Febrile neutropenia	0	0	13 (32.5%)	2 (5.0%)	15 (37.5%)
Blood bilirubin increase	8 (20.0%)	3 (7.5%)	1 (2.5%)	0	12 (30.0%)
Fatigue	4 (10.0%)	5 (12.5%)	0	0	9 (22.5%)
Diarrhoea	9 (22.5%)	0	0	0	9 (22.5%)
Nausea	6 (15.0%)	3 (7.5%)	0	0	9 (22.5%)
Creatinine increase	5 (12.5%)	0	0	0	5 (12.5%)
Headache	4 (10.0%)	0	0	0	4 (10.0%)
Vomiting	3 (7.5%)	1 (2.5%)	0	0	4 (10.0%)
Rash	4 (10.0%)	0	0	0	4 (10.0%)
Pruritus	2 (5.0%)	1 (2.5%)	0	0	3 (7.5%)
Pneumonia	0	0	3 (7.5%)	0	3 (7.5%)
Sepsis	0	0	2 (5.0%)	0	2 (5.0%)
Constipation	2 (5.0%)	0	0	0	2 (5.0%)
Dizziness	1 (2.5%)	0	0	0	1 (2.5%)
Anaemia	0	0	39 (97.5%)	1 (2.5%)	40 (100.0%)
Haematological adverse events					
Neutrophil count decrease	0	0	0	40 (100.0%)	40 (100.0%)
White blood cell count decrease	0	0	1 (2.5%)	39 (97.5%)	40 (100.0%)
Platelet count decrease	0	0	3 (7.5%)	37 (92.5%)	40 (100.0%)

## Discussion

This single-arm, phase 2 trial showed that the addition of chidamide to CAG plus venetoclax -azacitidine regimen could lead to a high ORR (97.5%) and CRc rate (85.0%) after one cycle of CACAG-VEN induction therapy in adults ≥60 years with newly diagnosed AML. Results from this study show that these drug combinations are well tolerated, with no early-death within 30days and promising clinical activity in terms of overall response and overall survival in patients aged ≥60 years with newly diagnosed AML.

The CACAG regimen showed a favorable response rates in the context of published studies on intensive chemotherapy. An ORR of 97.5%, including a composite CRc rate of 85.0%, was attained after one cycle of CACAG-VEN. The 3 + 7 regimen, using daunorubicin or idarubicin for 3 days and cytarabine for 7 days, has shown a complete remission rate of 70.0% in patients with good performance status, good organ function, and who do not have adverse cytogenetics (27). However, a number of AML patients over the age of 60 had poor performance status, or elevated bilirubin or creatinine levels which excluded them from conventional chemotherapy (6). The tolerability of the 3 + 7 regimen is limited in older and less fit patients. Based on its activity in combination with lower intensity chemotherapy, venetoclax has emerged as an important part of the standard of care for older or unfit patients with AML. For fit patients, adding venetoclax to modified IA regimen as the first line induction treatment of AML in patients aged ≥60 years showed CRc of 68% (in 28 patients) (28). In a study using VEN plus decitabine or azacitidine in treatment-naive, elderly patients with AML, the CRc rate after one cycle of the regimen was 67% (22). In contrast, our research found the high ORR and CRc rates across the ELN risk groups. (favorable 100.0% [ORR], 83.3% [CRc]; intermediate: 100.0% [ORR], 88.9% [CRc]; adverse: 93.8% [ORR], 81.3% [CRc]). In our subgroup analysis, the incidence of composite complete remission was notably improved across all AML genomic risk groups. We showed that patients with mutations in RUNX1, TET2, DNMT3A, or IDH1/2 who received CACAG-VEN induction therapy achieved a CRc rate of 100.0%. In particular, the TP53 mutation in AML is associated with poor prognosis (29). Four patients with TP53 mutations achieved CRc after undergoing CACAG-VEN induction therapy alone, of which 3 have been alive until the last follow-up, while one patient died of recurrence one year later. The response was favorable in TP53 mutant patients, highlighting efficacy in patients with poor prognosis. Therefore, a complete remission rate of 85.0% after one cycle of CACAG-VEN regimen indicates a stronger anti-leukemia activity of the regimen than that of conventional intensive chemotherapy.

In addition, the high rates of deep remission (with MRD-negativity) with the CACAG-VEN regimen could be observed in our cohort. After one cycle of the VEN plus decitabine or azacitidine regimen in elderly patients, the MRD-negative rates were only 28.0% (22). In our study, a high MRD-negative rate was observed in adverse-risk patients after one cycle of CACAG-VEN regimen (61.5%). In particular, after two courses of treatment with CACAG-VEN, a high MRD-negative rate was observed across the ELN risk groups. (favorable 100.0%; intermediate: 66.7%; adverse: 75.0%). Usually, the high rates of deep remission could potentially improve survival outcomes of patients with AML. Among these patients who received one cycle of CACAG-VEN, MRD-negative patients showed a trend of increased OS and decreased CIR compared to MRD-positive patients, albeit with non-significant statistical differences between the groups. Limitations of our study was that only 40 patients were included and the median follow-up duration was relatively short, so additional studies with more patients and a long-term follow-up are required.

One of the main concerns when adding venetoclax-azacitidine to intensive chemotherapy is the potential of increased adverse events. The most common grade 3–4 non-hematological adverse events were febrile neutropenia (37.5%), pneumonia (7.5%), sepsis (5.0%), and blood bilirubin increase (2.5%). These results were similar in frequency and intensity to rates reported in previous studies. In particular, the median time to recovery was 15.0 days for platelets ≥ 20000/μL and 13.0 days for an absolute neutrophil count ≥ 1000 cells/μL after the first cycle of the CACAG-VEN regimen, which were more rapid recovery times than in previous studies that showed a blood cell count recovery time of about 4 weeks (28, 30).

This treatment regimen not only targets the leukemia cells directly but also modulates the immune environment, which plays a crucial role in the overall response and survival of patients. Epigenetic manipulation has been reported to induce immune modulatory effects, which involve the heightened expression of tumor-associated antigens (31). The combination of these drugs can modulate the immune microenvironment in the bone marrow. Chidamide, in particular, has been shown to increase the expression of major histocompatibility complex (MHC) molecules on tumor cells and promote dendritic cell (DC) maturation, making them more visible to the immune system (32). This enhances the ability of cytotoxic T cells to recognize and kill leukemia cells.

To our knowledge, the regimen of chidamide in combined with CAG and venetoclax-azacitidine demonstrated promising efficacy in elderly patients with AML, with a high ORR rate (97.5%) and CRc rate (85.0%). The CACAG-VEN regimen was well tolerated, with no early-death within 30days and short duration of pancytopenia. A well designed randomized trial with long-term follow-up is now warranted.

## Data Availability

The original contributions presented in the study are included in the article/**Supplementary Material**. Further inquiries can be directed to the corresponding authors.
