# Supplementary material for: Phase 2 study of chidamide in combination with CAG and venetoclax-azacitidine in older patients with newly diagnosed acute myeloid leukemia
Source: Front Immunol. 2025 Feb 26;16:1525110. doi: 10.3389/fimmu.2025.1525110 (PMC11897025; doi:10.3389/fimmu.2025.1525110)
Supplement: Supplementary file 1 [file DataSheet1.docx]

**1 Supplementary Methods**

**1.1 Patients and Procedures**

During treatment, symptomatic and supportive therapies were administered to protect liver and kidney function (hydration and alkalinization) while preventing tumor lysis syndrome and infection. If patients needed initiation of antifungal treatment or other moderate or strong CYP3A4 inhibitors, the venetoclax dose was adjusted according to the recommended prescription information.

The bone marrow was evaluated on day 28 after the start of therapy. Measurable residual disease (MRD) was tested by flow cytometry assessment and bone marrow cytology during each treatment cycle. Samples with at least 100,000 cells were considered evaluable. Baseline and scheduled samples were collected for genomic sequencing. Cardiac function assessments, as well as regular tests such as biochemistry, electrocardiography, and echocardiography, monitored cardiac, renal, and hepatic functions were performed at the physician’s discretion. Adverse events were assessed according to standard criteria, with continuous monitoring throughout the study.

**1.2 Endpoints**

CR was defined as bone marrow with less than 5% blasts, a platelet count >100,000/mm^3^, red blood cell transfusion independence, and an absolute neutrophil count >1,000 cells/mm^3^. CRi was defined as all of the criteria for CR, except for neutropenia (absolute neutrophil count ≤1,000/mm^3^) or thrombocytopenia (platelet count ≤100,000/mm^3^). PR was defined for all hematologic criteria of CR, decreased bone marrow blast percentage to 5%–20%, and decreased pretreatment bone marrow blast percentage by at least 50%. The MRD was evaluated with a sensitivity of 0.01%–0.10%, defining MRD negativity as <0.1% and MRD positivity as ≥0.1%. OS was defined as the time interval from treatment initiation to the date of death from any cause. EFS was defined as the time from enrollment to the occurrence of any event, including disease progression, cessation of treatment for any reason, or death. DOR was defined among responders as the duration between the date of response and the date of disease relapse or death from any cause, whichever occurred first. CIR was defined as the incidence of relapse after CRc, with death in CRc as a competing risk.

**Table S1. Eligibility Criteria**

| Inclusion Criteria:   1. Voluntary participation in the clinical study; the patient himself/herself or his/her legal guardian fully understands, is informed of the study and signs the ICF; is willing to follow and can complete all trial procedures. 2. Patients were 60 years of age or older at enrollment, regardless of gender. 3. Patients who are newly diagnosed with AML (excluding APL, including secondary AML). 4. No severe allergies. 5. Liver function: ALT and AST ≤ 2.5 × ULN, bilirubin ≤ 2 × ULN. 6. Kidney function: creatinine ≤ ULN. 7. No uncontrollable infections or serious psychiatric disorders. 8. The score of Eastern Cooperative Oncology Group is ≤ 2. |
| --- |
| Exclusion Criteria   1. Patients with allergies and contraindications to the study drug. 2. Pregnant and lactating female patients. 3. Patients with active infections. 4. Patients with a known history of alcohol or drug addiction.   (5) Patients with mental illness or other conditions unable to comply with the protocol.  (6) Patients who have not completed 6 weeks after surgery on vital organs of the body.  (7) Renal function: creatinine > ULN  (8) Not suitable for this clinical trial in the opinion of the investigator (e.g. poor  compliance, drug abuse, etc.). |

ULN, the upper limit of normal; APL, acute promyelocytic leukemia; AML, acute myeloid leukemia; ICF, informed consent form.

**Table S2. Patients demographics**

| Pt# | Age(Yrs) | Gender | ECOG | FAB | 2022 ELN risk status | Molecular | Baseline parameters | | | |
| --- | --- | --- | --- | --- | --- | --- | --- | --- | --- | --- |
|  |  |  |  |  |  |  | WBC | Hb | Plt | BM  blasts(%) |
| 1 | 69 | M | 2 | M5 | Adverse | TP53/P278R | 34.6 | 93 | 151 | 91.0 |
| 2 | 68 | F | 1 | M2 | Intermediate | / | 4.1 | 71 | 41 | 24.0. |
| 3 | 60 | M | 1 | M5 | Adverse | MYC/FLT3/MPL/KRAS/SRSF2/NPM1/TET2 | 27.97 | 44 | 23 | 74.0 |
| 4 | 64 | M | 0 | / | Favorable | / | 2.91 | 77 | 18 | 17.3 |
| 5 | 65 | M | 1 | M4 | Adverse | CBL/ASXL1/TET2/STAG2 | 118.05 | 81 | 57 | 25.2 |
| 6 | 64 | F | 0 | / | Intermediate | / | 1.22 | 101 | 108 | 46.8 |
| 7 | 65 | M | 1 | / | Intermediate | NPM1 | 46.12 | 67 | 52 | 46.15 |
| 8 | 66 | M | 1 | M5 | Intermediate | DNMT3A/IDH1 | 26.39 | 108 | 254 | 90.0 |
| 9 | 60 | M | 0 | / | Adverse | U2AF1/WT1 | 2.76 | 66 | 111 | 23.2 |
| 10 | 61 | F | 1 | M2/M4 | Intermediate | NPM1 | 2.5 | 125 | 208 | 78.0 |
| 11 | 66 | M | 1 | / | Favorable | NPM1/DNMT3A/IDH2/NRAS | 16.9 | 69 | 86 | 48.0 |
| 12 | 60 | M | 0 | M2 | Intermediate | CSF3R/KIT D816 | 29.09 | 93 | 67 | 80.0 |
| 13 | 74 | F | 0 | M4 | Intermediate | KRAS/NRAS | 2.61 | 58 | 32 | 21.5 |
| 14 | 73 | F | 1 | M5 | Adverse | ASXL1/RUNX1/EZH2/PTPN11 | 21.23 | 94 | 78 | 32.4 |
| 15 | 60 | F | 2 | M4/M5 | Adverse | TP53/JAK2/V617F | 7.24 | 53 | 34 | 55.0 |
| 16 | 63 | M | 1 | M4/M5 | Favorable | NPM1 | 3.27 | 68 | 27 | 70.0 |
| 17 | 60 | M | 0 | / | Adverse | NRAS/PTPN11/U2AF1/ASXL1 | 21.52 | 112 | 196 | 30.8 |
| 18 | 69 | F | 1 | / | Adverse | STAG2/KMT2A | 2.16 | 84 | 199 | 23.2 |
| 19 | 66 | M | 0 | / | Intermediate | / | 2.21 | 2.95 | 104 | 50.0 |
| 20 | 64 | M | 1 | / | Intermediate | NPM1/IDH2/FLT-ITD | 58.7 | 163 | 88 | 87.2 |
| 21 | 66 | F | 1 | M5 | Adverse | / | 4.5 | 53 | 68 | 53.0 |
| 22 | 60 | F | 1 | M4 | Adverse | TP53/CEP17 | 27.46 | 59 | 39 | 60.0 |
| 23 | 60 | M | 1 | M4 | Adverse | / | 20.37 | 63 | 87 | 52.0 |
| 24 | 72 | F | 2 | M5 | Intermediate | / | 35.5 | 2.84 | 91 | 72.0 |
| 25 | 60 | M | 1 | / | Intermediate | / | 1.82 | 73 | 173 | 91.2 |
| 26 | 66 | M | 0 | M2 | Intermediate | WT1 | 1.82 | 80 | 42 | 32.0 |
| 27 | 67 | F | 2 | M2 | Intermediate | DDX41 | 1.53 | 84 | 31 | 28.5 |
| 28 | 60 | M | 0 | / | Adverse | CSF3R/SRSF2/CEBPA/ASXL1/TET2/RUNX1 | 176.4 | 68 | 31 | 22.4 |
| 29 | 74 | M | 1 | / | Intermediate | / | 1.36 | 55 | 82 | 57.6 |
| 30 | 67 | M | 1 | M2 | Intermediate | CBL/SETBP1/DDX | 1.5 | 111 | 51 | 19.6 |
| 31 | 62 | F | 0 | / | Adverse | ASXL1 | 8.94 | 14 | 112 | 23.0 |
| 32 | 60 | M | 1 | / | Adverse | BCOR、RUNX1 | 1.82 | 289 | 95 | 36.0 |
| 33 | 70 | M | 2 | / | Adverse | EZH2、TP53、DNMT3A | 17.29 | 43 | 57 | 50.0 |
| 34 | 65 | F | 2 | M5 | Adverse | DNMT3A、SETBP1 | 31.64 | 107 | 89 | 93.2 |
| 35 | 61 | M | 1 | M2 | Intermediate | KIT D816 | 5.7 | 5 | 90 | 88.0 |
| 36 | 62 | F | 1 | M4/M5 | Intermediate | / | 26.48 | 17 | 96 | 90.0 |
| 37 | 62 | M | 1 | / | Favorable | / | 63.2 | 45 | 96 | 64.8 |
| 38 | 62 | F | 0 | M4 | Favorable | DNMT3A、NPM1 | 6.52 | 60 | 88 | 86.4 |
| 39 | 63 | M | 0 | M2 | Favorable | KIT D816、DHX15 | 7.89 | 16 | 75 | 76.0 |
| 40 | 62 | M | 1 | / | Intermediate | IDH1、NPM1、FLT-ITD | 18.49 | 36 | 115 | 85.6 |

**Table S3. Response after two cycle of the CACAG-VEN regimen (n= 26)**

| Overall response rate | Patients (n=26*) | Patients (n=26) | | |
| --- | --- | --- | --- | --- |
|  |  | Favourable Risk*  (n=4) | Intermediate Risk*  (n=9) | Adverse Risk*  (n=13) |
| ORR % ( 95%CI) | 100.0 (84.0-100.0) | 100.0 (39.6-100.0) | 100.0 (62.9-100.0) | 100.0 (71.6-100.0) |
| CR or CRi, n (%, 95%CI) | 25 (96.2, 78.4-99.8) | 4 (100.0, 39.6-100.0) | 9 (100.0, 62.9-100.0) | 12 (92.3, 62.1-99.6) |
| CR, n (%, 95%CI) | 14 (53.9, 33.8-72.9) | 4 (100.0, 39.6-100.0) | 5 (55.6, 22.7-84.7) | 5 (38.5, 15.1-67.7) |
| CRi, n (%, 95%CI) | 11 (42.3, 24.0-62.8) | 0 (0.0, 0.0-60.4) | 4 (44.4, 15.3-77.3) | 7 (53.9, 26.1-79.6) |
| PR, n (%, 95%CI) | 1 (3.9, 0.1-21.6) | 0 (0.0, 0.0-60.4) | 0 (0.0, 0.0-37.1) | 1 (7.7, 0.1-37.9) |
| NR, n (%, 95%CI) | 0 (0.0, 0.0-16.0) | 0 (0.0, 0.0-60.4) | 0 (0.0, 0.0-37.1) | 0 (0.0, 0.0-28.3) |
| MRD-negative rate in patients with response (95%CI) | 76.0 (19/25, 54.5-89.8) | 100.0 (4/4, 39.6-100.0) | 66.7 (6/9, 30.9-91.0) | 75.0 (9/12, 42.8-93.3) |

CR, complete response; CRi, CR along with incomplete blood count recovery; PR, partial response ; NR, no response; ORR, overall response; *patients were stratified based on ELN (2022) risk assessment criteria; CACAG-VEN, venetoclax combined with chidamide, azacitidine, cytarabine, aclarubicin, and granulocyte colony-stimulating factor.

**Table S4. Hematopoietic recovery and surpportive treatment during first cycle of therapy**

| Hematopoietic recovery and surpportive treatment | |
| --- | --- |
| Days to absolute neutrophil count recovery to ≥1 000 cells per µL, median (IQR) | 15.0 (10.0-19.5) |
| Days to absolute platelets count recovery to ≥20 000 per µL, median (IQR) | 13.0 (10.5-17.0) |
| Packed red cells units, median (range) | 8.5 (2.0-23.5) |
| Platelet units, median (range) | 8.5 (2.0-19.5) |

IQR, inter-quartile range.


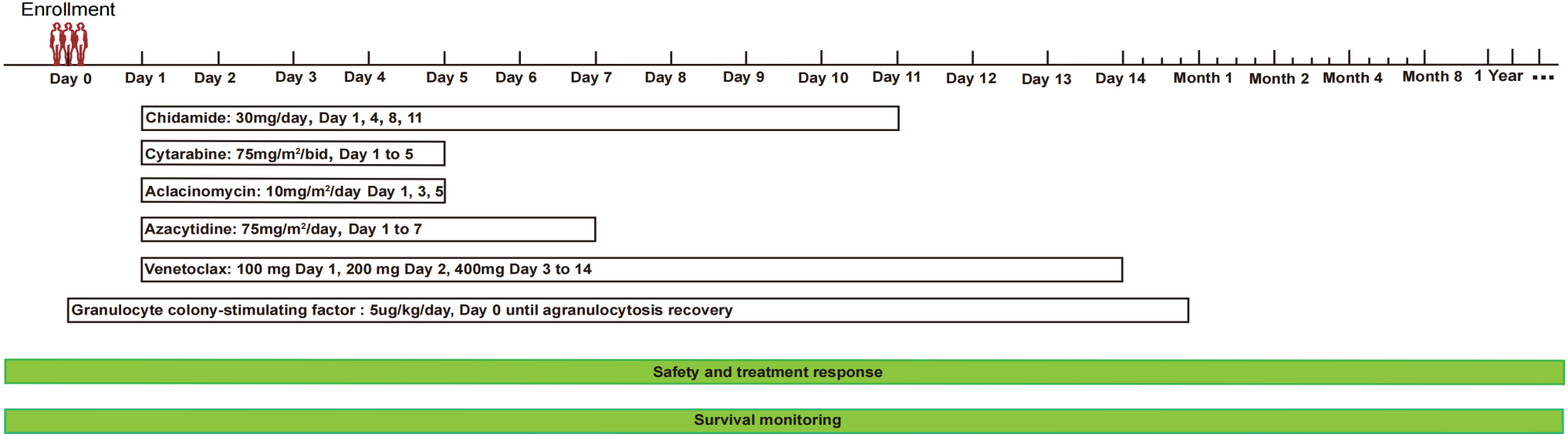


Figure S1. Treatment schema of CACAG-VEN. CACAG-VEN, venetoclax combined with chidamide, azacitidine, cytarabine, aclarubicin, and granulocyte colony-stimulating factor as induction treatment; bid, twice a day; Day, the time after enrollment.


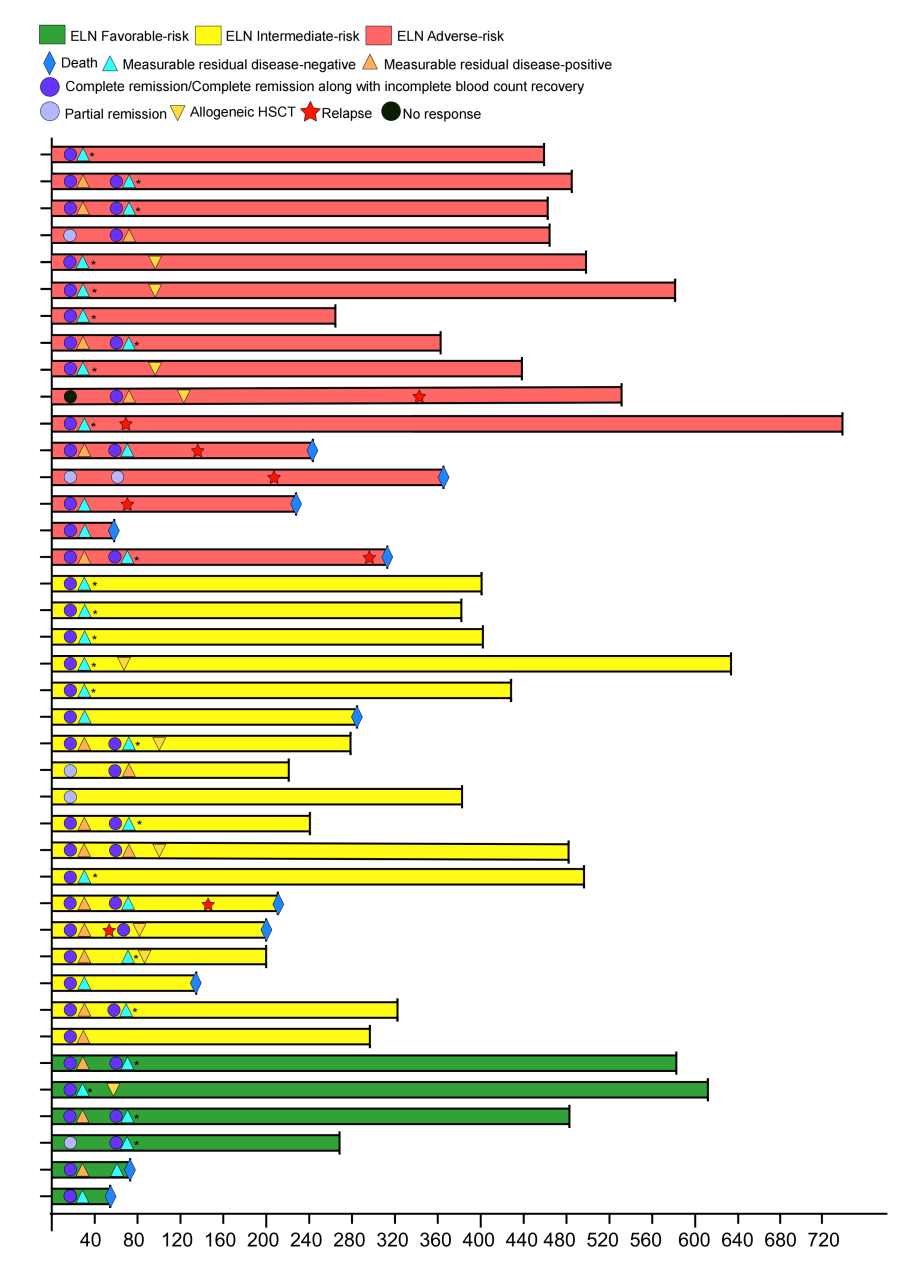


Figure S2. Swimmer plot of dynamic response assessment. Each bar represents an individual patient; HSCT=haematopoietic stem-cell transplantation. *Continuous measurable residual disease-negativity.


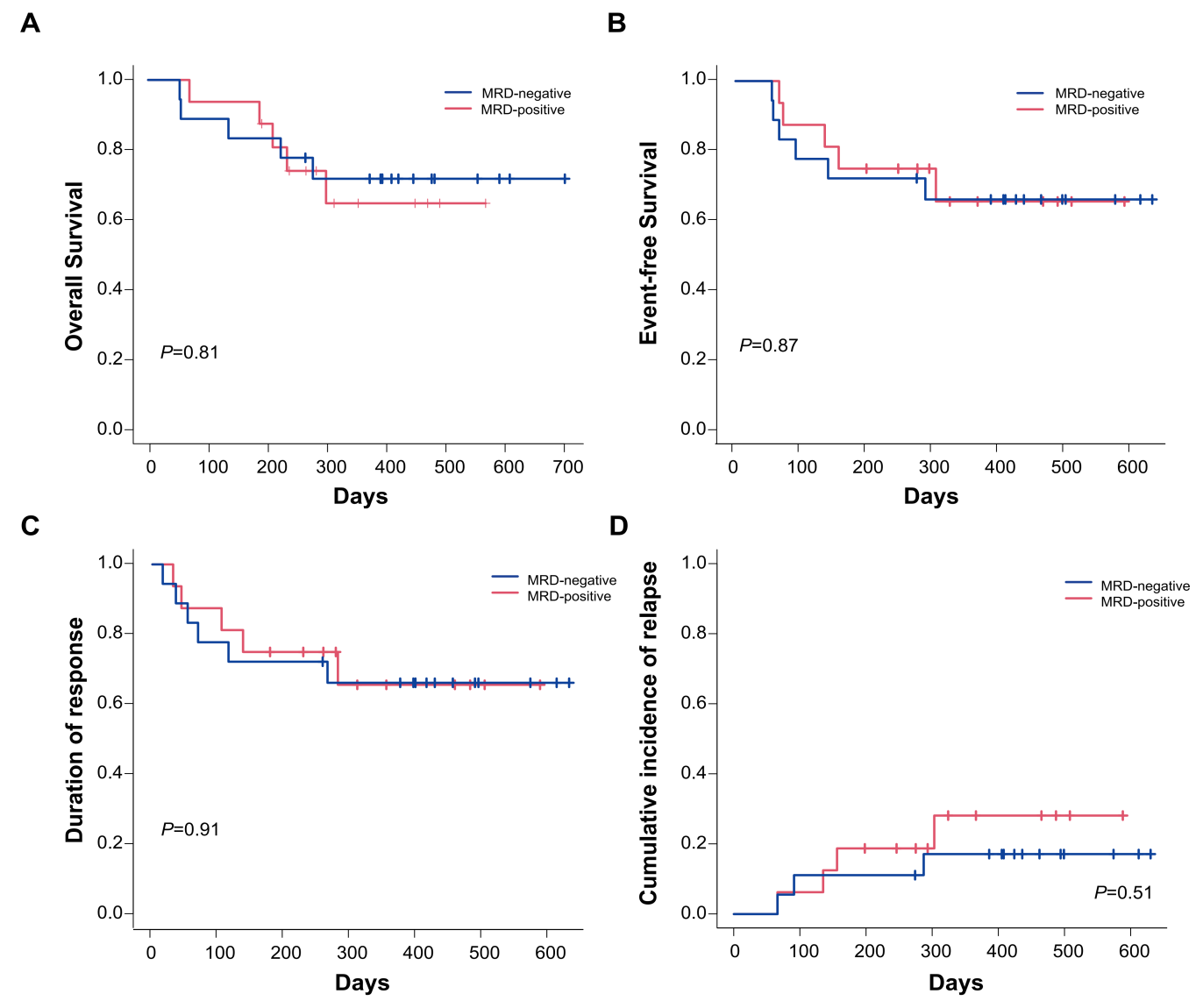


Figure S3. Cumulative incidence of OS A), EFS (B), DOR (C), and CIR (D) in the patients received one cycles of CACAG-VEN regimen, grouped by MRD status. OS, overall survival; EFS, event-free survival; DOR, duration of response; CIR, cumulative incidence of relapse; MRD, measurable residual disease.


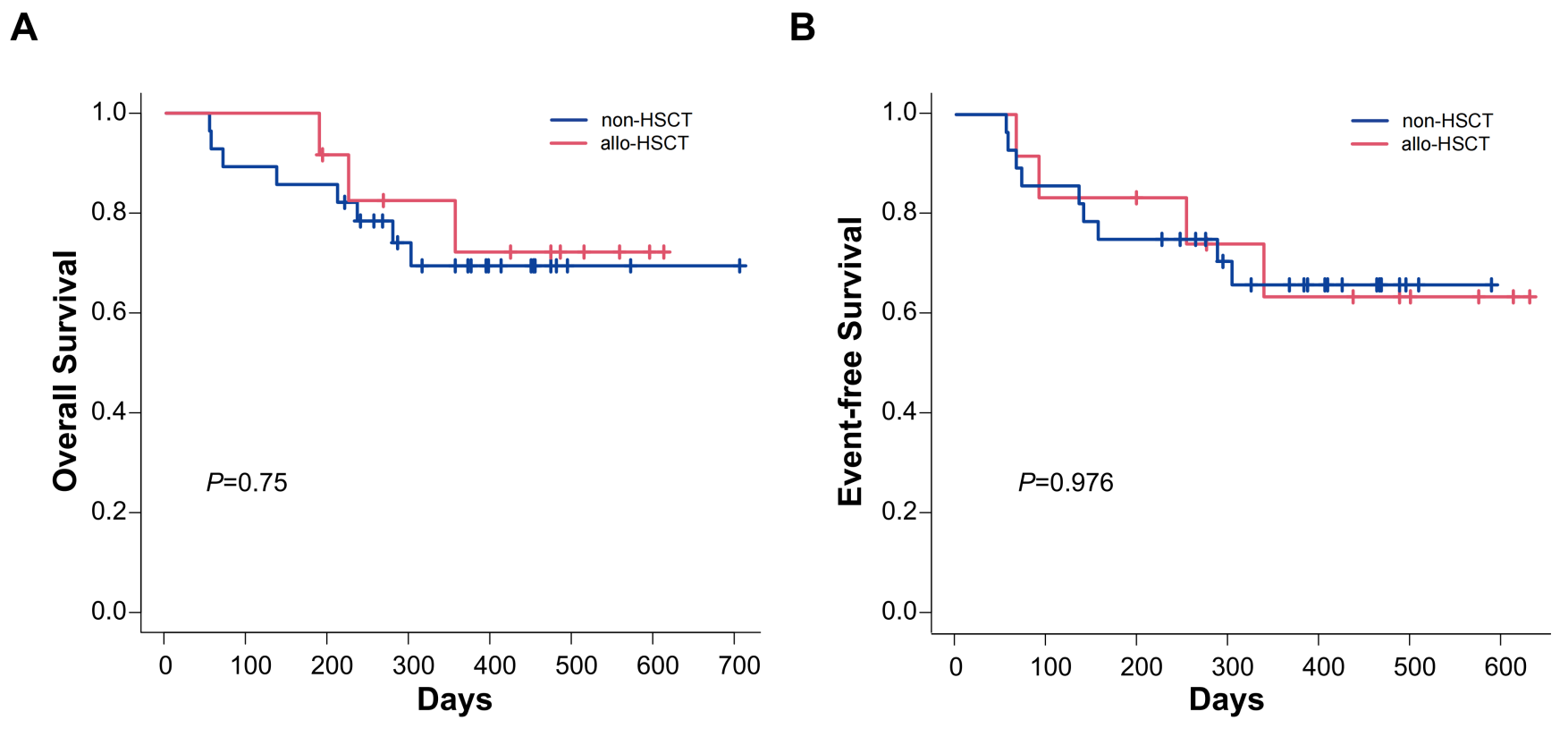


Figure S4. Cumulative incidence of OS A), EFS (B) in the patients received one cycles of CACAG-VEN regimen, grouped by allo-HSCT. OS, overall survival; EFS, event-free survival.
